# Altered parasympathetic outflow and central sensitization response to continuous pain in cyclic vomiting syndrome: a functional magnetic resonance imaging study

**DOI:** 10.1152/ajpgi.00011.2024

**Published:** 2024-11-15

**Authors:** Andrew Bolender, Rowan Staley, Ronald G. Garcia, Riccardo Barbieri, Ovidiu Andronesi, Shahar Castel, Andrea Thurler, Vitaly Napadow, Braden Kuo, Roberta Sclocco

**Affiliations:** 1 Athinoula A. Martinos Center for Biomedical Imaging, Department of Radiology, Massachusetts General Hospital, Harvard Medical School, Charlestown, Massachusetts, United States; 2 Department of Gastroenterology and Center for Neurointestinal Health, Massachusetts General Hospital, Harvard Medical School, Boston, Massachusetts, United States; 3 Department of Psychiatry, Massachusetts General Hospital, Harvard Medical School, Boston, Massachusetts, United States; 4 School of Medicine, Universidad de Santander, Bucaramanga, Colombia; 5 Department of Electronics, Information and Bioengineering, Politecnico di Milano, Milano, Italy; 6 Department of Physical Medicine and Rehabilitation, Spaulding Rehabilitation Hospital, Harvard Medical School, Charlestown, Massachusetts, United States

**Keywords:** cyclic vomiting syndrome, interoception, neuroimaging, temporal summation of pain

## Abstract

Cyclic vomiting syndrome (CVS) is a disorder of brain-gut interaction characterized by recurrent episodes of nausea and vomiting interspersed with asymptomatic periods and associated with autonomic nervous system dysfunction. We examined the dysautonomic response to noxious stimuli for patients with CVS using our previously validated approach to integrate peripheral autonomic outflow metrics, temporal summation of pain, and brain functional MRI (fMRI). Blood oxygen level-dependent (BOLD) fMRI and ECG were acquired from patients with CVS and healthy adults during both a rest condition and a sustained cuff pressure-pain stimulus at the leg. After the latter scan, participants rated pain for the first, middle, and last 2 min to calculate temporal summation. During sustained pain, patients, relative to healthy controls, exhibited greater reduction in heart rate variability within the high-frequency range (HF-HRV) and reduced anticorrelation between HF-HRV and fMRI signal in the anterior insula, pregenual anterior cingulate cortex, and ventrolateral and dorsolateral prefrontal cortex. Compared with healthy adults, patients also exhibited increasing pain intensity over the course of sustained cuff pressure. For healthy adults, seed-based functional connectivity analysis revealed pain sensitization correlated with pain-induced increases in connectivity between primary somatosensory cortex and regions of interest in both left anterior insula/posterior orbitofrontal cortex and right presupplementary motor area. These correlations were not seen in CVS, thus supporting a conclusion of altered central coding of nociceptive stimuli and autonomic responsivity of patients with CVS in key brain regions implicated in autonomic control and interoception.

**NEW & NOTEWORTHY:**

Patients with cyclic vomiting syndrome exhibit multiple alterations in central function in response to a sustained pressure-pain stimulus, including altered high-frequency heart rate variability and associated changes in BOLD fMRI signal in key areas of the central autonomic and interoceptive networks, as well as abnormal temporal summation of pain associated with altered connectivity patterns between the primary somatosensory cortex and key regions associated with interoception.

## INTRODUCTION

Cyclic vomiting syndrome (CVS) is a debilitating disorder of brain-gut interaction characterized by recurrent episodes of intense nausea and vomiting interspersed with asymptomatic periods of varying duration. CVS is considered a disorder of the central nervous system with etiology similar to that of migraines or epilepsy, despite primary evaluation by gastroenterologists. With treatments ranging from pharmaceuticals like sumatriptan to interventions like hot showers, clinical management prioritizes decreasing the symptom profile once an episode is triggered by an aversive environmental stimulus like psychological or physical stress, sleep disruption, or an adverse dietary reaction (1–3).

Although the underlying pathophysiology of CVS is poorly understood, previous research suggests that, like migraine, symptoms may arise from a confluence of multiple mechanisms (4). Implicated among these is multinodal dysfunction of the autonomic nervous system, including sympathovagal response to noxious stressors: pediatric CVS patients exhibit aberrant cardiac vagal regulation compared with healthy children (5, 6), and a large subset of these patients develop comorbid autonomic dysfunction during adolescence (7). These comorbidities suggest a mechanism where neurological abnormalities in autonomic processing may contribute to both the CVS phenotype and other autonomic pathologies. The exploration of the autonomic and central mechanisms at play in CVS pathophysiology is critical for the development of effective treatment plans and may offer opportunities for prophylactic interventions, for which current evidence is largely low quality according to consensus guidelines (8).

Neuroimaging studies investigating brain-gut axis dysfunction underlying CVS remain scarce, but they may begin to elucidate some central pathophysiology. For example, our previous research comparing CVS with migraine and healthy adults suggested the central role of the insula, a key region associated with interoception and viscerosensory integration. Both CVS and episodic migraine are characterized by decreased connectivity, the degree of synchronicity in activity over time between brain regions, between the posterior insula and the sensorimotor network, suggesting an abnormal decoupling of viscerosensory processing in these areas for both conditions (9). However, patients with CVS exhibit an increased connectivity between the salience network and the mid/posterior insula compared with both patients with migraine and healthy controls, indicating both unique and shared pathology between CVS and migraine (9).

We can use functional MRI (fMRI) to characterize the networks involved in autonomic processing by evaluating the correlation between time-varying fMRI activity and metrics for autonomic nervous system activity, such as the power of high-frequency heart rate variability (HF-HRV), a proxy for parasympathetic outflow. Our work exploring the central mechanisms of nausea using this approach revealed an increase in heart rate (HR) and a decrease in HF-HRV during nausea, alongside alterations in the central activity associated with HF-HRV (10). These results suggest that nausea is associated with decreased parasympathetic activity and a switch in the central processing of that activity. Underlying neurological abnormalities may prime CVS patients to have hypersensitivity to autonomic stressors, which might produce measurable alterations in brain activity associated with autonomic activity during perturbation.

One approach to examining the autonomic nervous system’s response to stressors is the introduction of a noxious pain stimulus. Application of continuous or repeated pain stimuli has been used to interrogate autonomic dysfunction in multiple chronic pain disorders (11–13). We have previously applied a sustained pain challenge in patients with chronic pain to investigate pathological disruptions in central autonomic processing and functional connectivity between the somatosensory cortex and other brain regions (14). Given the implications of central autonomic dysfunction in CVS and the overlap of key brain regions associated with nausea and pain pathways (15–17), we sought to combine these methods of mapping brain regions involved in autonomic processes and probing the autonomic nervous system with a sustained pressure-pain challenge to interrogate potential central autonomic alterations involved in CVS pathophysiology.

Our current study applied a novel approach integrating peripheral autonomic recordings with whole brain fMRI data to investigate the central autonomic network during a sustained noxious stimulus in CVS as compared with healthy adults. In this study, we synchronously collected brain and parasympathetic outflow metrics with blood oxygen level-dependent (BOLD) fMRI and electrocardiogram (ECG), respectively. We hypothesized that patients with CVS would exhibit altered central activity associated with HF-HRV and pain perception during a sustained pressure-pain stimulus.

## MATERIALS AND METHODS

### IRB Statement

The protocol was approved by the Human Research Committee of Mass General Brigham, and all experiments took place at the Athinoula A. Martinos Center for Biomedical Imaging in Charlestown, MA. All participants provided written, informed consent in accordance with the policies of the Human Research Committee at Massachusetts General Hospital prior to participation.

### Participants

Our sample consisted of 14 patients with CVS (12 females; mean age ± SD = 29.21 ± 11.67 yr) and 14 sex- and age-matched healthy adults (12 females; mean age ± SD = 29.29 ± 11.08 yr). All participants were enrolled between 2014 and 2015, right-handed, and English speaking. Our inclusion criteria for CVS were age 18–80 with a diagnosis of CVS by Rome III criteria (including acute onset episodes of vomiting of duration <1 wk, 3 or more discrete episodes in the prior year, and absence of nausea and vomiting between episodes). Of note, we enrolled neuromodulatory pharmacotherapy-naïve CVS patients to reduce the potential effects of such pharmacotherapy on functional brain imaging outcomes. Due to the intensity of CVS episodes and the possibility of emesis inside the MRI scanner, all patients with CVS were in an interictal state (95% confidence interval [CI: 41.2–239.8] days since the previous episode) during the study visits, with a minimum of 48 h after any nausea/vomiting incident. Patients with CVS with a history of cannabinoid usage were eligible provided they refrained from use for 7 days (cannabis negative) before visits, to reduce confounds due to the similar presentation of cannabinoid hyperemesis syndrome to CVS. The inclusion criterion for healthy adults was age 18–80 yr. Exclusion criteria included serious medical illness (kidney failure, congestive heart failure, and diabetes), pregnancy, use of benzodiazepines within 7 days, opioid use, other neurological or major psychiatric conditions, as well as any MRI contraindications such as metallic implants. Participants were instructed to abstain from alcohol for 24 h prior to testing and refrain from smoking or caffeine consumption on the day of the visit.

### Experimental Setup

**Figure 1.**
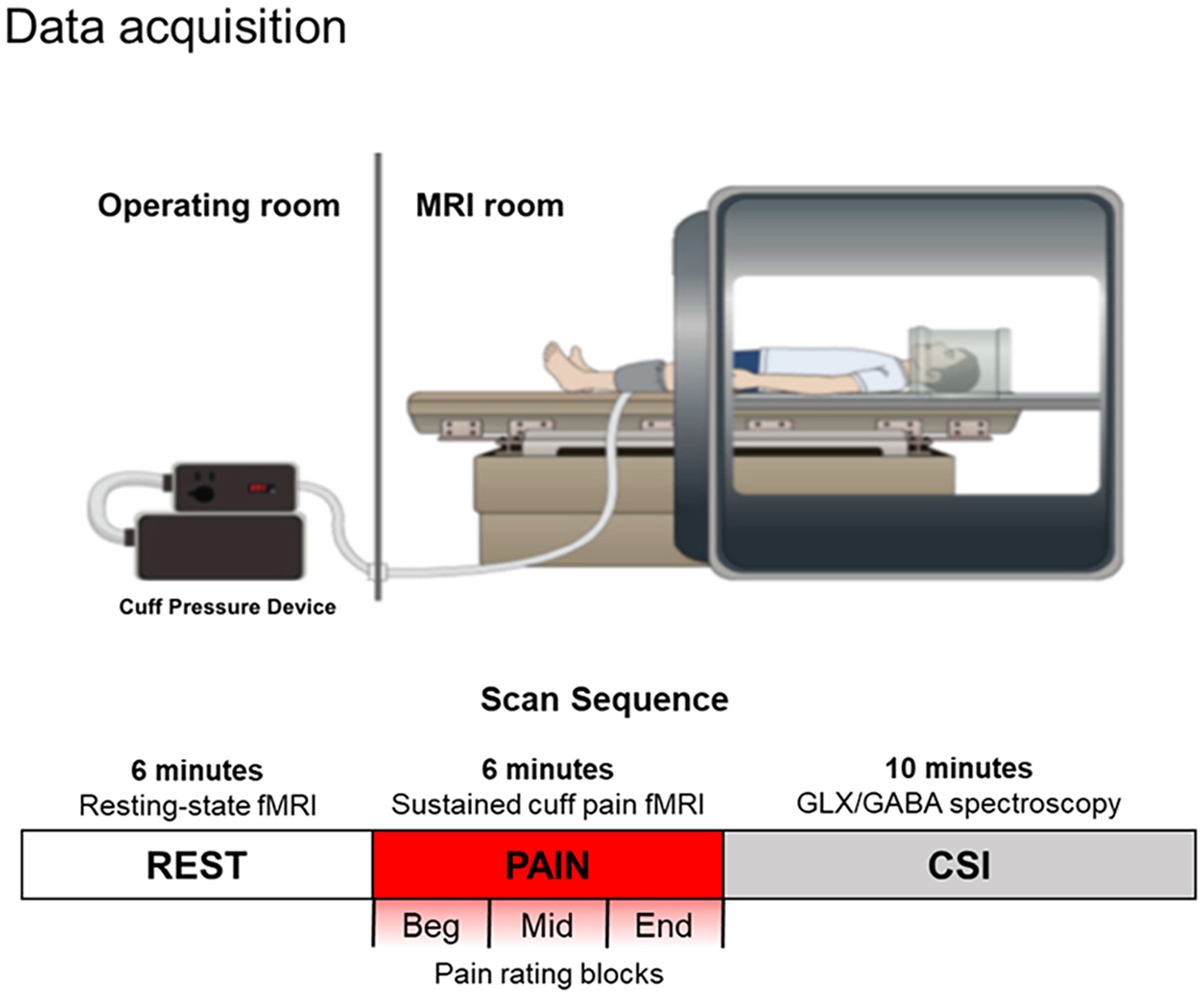
Schematic diagram of the experimental setup and collected data. Autonomic nervous system and fMRI data were concurrently acquired during the 6-min REST and PAIN sequences, followed by Chemical Shift Imaging (Glx/GABA) data. fMRI, functional MRI.

Participants were placed supine in a 3T Siemens Tim Trio MRI Scanner (Siemens Medical Systems, Erlangen, Germany) using a 32-channel head coil (Fig. 1). Whole brain BOLD fMRI data were collected using a gradient echo T2*-weighted pulse sequence (repitition time (TR) = 2500 ms, echo time = 30 ms, 43 slices, slice thickness = 2.6 mm, matrix = 84 × 84, flip angle = 90°). The fMRI images were collected continuously during a 6-min baseline resting state (REST) condition and a 6-min continuous noxious stimulus (PAIN) condition designed to challenge the autonomic nervous system. The noxious stimulus consisted of an adjustable pressure cuff (SC12D; Hokanson Inc., Bellevue, WA) placed around the participants’ left gastrocnemius muscle that was connected to a rapid cuff inflator (Hokanson E20 AG101). When inflated, the cuff delivered painful sensation individually calibrated at a prior behavioral visit, as matched by a pain intensity rating of 40 out of 100 (anchors of “no pain” and “intolerable pain”). After the PAIN run, participants were asked to rate *1*) the overall intensity of pain throughout the run, followed by *2*) the intensity of pain from the cuff for each of the 2-min blocks at the beginning, middle, and end of the 6 min, to retrospectively evaluate potential sensitization or habituation to the continuous pain stimulus. The order of the two fMRI runs was not counterbalanced across participants to avoid potential lingering pain from the noxious stimulus during REST.

Peripheral physiological signals, including heartbeat and respiration, were collected at 400 Hz using a 16-channel PowerLab DAQ System (ADInstruments, Colorado Springs, CO) on a laptop equipped with Chart Data Acquisition Software (ADInstruments). MRI-compatible electrodes (VerMed, Bellows Falls, VT) were placed on the participants’ chest to collect electrocardiogram (ECG), and respiration was measured by a custom-built pneumatic belt placed around the participants’ abdomen. Low compliance tubing connected the belt to a pressure transducer (PX138-0.3D5V; Omegadyne, Inc., Sunbury, OH), and the voltage signal reflecting respiratory volume was recorded.

Magnetic resonance spectroscopic imaging (MRSI) was performed using our three-dimensional Mescher-Garwood-difference-edited pulse sequence with localized adiabatic spin-echo refocusing (MEGA-LASER), spiral spatial-spectral encoding, and real-time motion correction and shim update. This sequence was previously validated for in vivo mapping of GABA (18). Scans were executed with the following parameters: TR = 1,600 ms, 3 mL isotropic voxels, 14 × 14 × 12 matrix, field of view 200 × 200 × 120 mm, volume of interest 110 × 90 × 50 mm, bandwidth 1.25 kHz, 10 weighted averages, four preparation scans, acquisition time TA 12:50 min:s, and spectral editing of the GABA signal at 3 parts per million (18). We have presented MRSI results from this MEGA-LASER sequence in a prior publication (19). Spectroscopy sequences were finalized after recruitment was partially complete; thus, neurochemical measures were carried out on a reduced sample size of 6 patients with CVS and 10 healthy adults.

### ECG Processing

Time-varying autonomic metrics of heart rate variability (HRV) were calculated from annotated ECG waveforms via a point-process algorithm using local likelihood estimation of instantaneous heart rate (HR) dynamics, focusing on the high-frequency component, 0.15–0.40 Hz, of the HRV spectrum (HF-HRV, a measure of parasympathetic activity) (20, 21). This algorithm better captures the point process of heartbeats, has been cross-validated with traditional time-frequency domain methods (20), and provides improvements in measuring fast, dynamic heart rate changes for instantaneous HRV estimation compared with traditional root-mean-square models (22). To evaluate pain-induced changes in autonomic function between groups, absolute HF-HRV power was averaged over sessions and across participants for REST and PAIN conditions, and then the relative change from REST, that is, (PAIN – REST)/REST × 100, was calculated. To integrate HF-HRV with fMRI data, dynamic HF-HRV indices were low-pass filtered at 0.33 Hz and downsampled at fMRI TR time points. The HF-HRV was further thresholded at the 98th percentile and normalized between 0 and 1 as in previous work (10, 23). HR was convolved with the cardiac response function, which can correct the BOLD signal for alterations from HR, and used as a confound in subsequent analyses (24). One CVS and one healthy adult participant had poor ECG signal quality during PAIN scans and were removed from HF-HRV and fMRI analyses. Two additional CVS participants had poor ECG signals during rest scans and were removed from the functional connectivity analysis.

### Temporal Summation Index

The retrospective ratings 0–100 on the numeric rating scale (NRS) for each of the 2-min subsections of the 6-min fMRI run (beginning, middle, and end) were used to calculate a temporal summation of the pain index reflective of the relative change in pain rating over time. Specifically, we calculated the temporal summation index by subtracting the beginning rating from the end rating, divided by the beginning rate to assess sensitization to a constant pain stimulus. We did not incorporate the individually variable cuff pressure (mmHg) into the calculation because *1*) pain perception was the primary outcome measure and *2*) we found no significant difference in cuff pressure between groups (healthy adults: 202.29 ± 81.03 mmHg; CVS: 222.86 ± 92.40 mmHg; *P* = 0.52).

### fMRI Data Preprocessing

fMRI BOLD data preprocessing was performed using a combination of FSL (v6.0.4), AFNI, and in-house scripts. Preprocessing steps included physiological noise correction using RETROICOR, motion correction, brain extraction, high-pass filtering (*f* > 0.007 Hz), spatial smoothing (full-width at half-maximum = 5 mm), and spatial normalization to Montreal Neurological Institute (MNI) space.

### HF-HRV/fMRI Analysis

We sought to evaluate central processing of cardiovagal output proxied by time-varying HF-HRV. To that end, first-level, whole brain general linear model (GLM) analyses were performed for each participant and fMRI was run using FSL Feat (v6.0.4). For each scan run, time-varying HF-HRV indices were convolved with a canonical hemodynamic response function and used as regressors of interest in the GLM. Additional regressors of no interest included instantaneous HR convolved with the cardiac response function to correct for BOLD signal noise related to instantaneous HR and motion outliers (FSLMotionOutliers, threshold = 75th percentile + 1.5 × interquartile range) (24). Single-participant parameter estimates were then passed up to group-level analysis. CVS (*n* = 13) and healthy adult participants (*n* = 13) were compared during the fMRI sustained cuff pain condition (PAIN) (FLAME1 + 2, FEAT, FSL). Statistical significance was set at *Z* > 2.3 with a corrected cluster significance threshold of *P* < 0.05. Significant clusters spanned multiple functional regions, so spherical regions of interest (radius 4 mm; ROIs) centered at local Z-statistic maxima and masked by significant clusters were generated. The average signal within each spherical ROI was extracted from each participant’s first-level map for visualization. ROI functional region names were identified using the Harvard-Oxford cortical structural atlas, the FSL Cerebellar Atlas with Nonlinear Registration, and investigator identification (25, 26). See Table 1 for participant characteristics.

### Pain Response Analysis

To examine alterations in pain perception, mixed ANOVA was performed evaluating the effects of independent variables Group (CVS *n* = 14, healthy adults *n* = 14, Table 2; between participants) and time block within the sustained pain scan (beginning, middle, and end; within participants) on dependent variable pain intensity rating, followed by post hoc paired *t* tests evaluating the significance of differences in pain intensity ratings between time blocks within each group. Statistical tests were performed in RStudio v2022.07.2 (PBC, Boston, MA). See Table 2 for participant characteristics.

### Seed-Based Functional Connectivity Analysis

To investigate whether variations in temporal summation were linked to changes in central processing of the cuff inflation stimulus, we performed a second GLM analysis evaluating whole brain functional connectivity, a measure of synchronicity in activity, with the primary somatosensory cortex, that is, the degree to which BOLD activity over time in the primary somatosensory cortex correlated with activity throughout the rest of the brain. We utilized a seed for the primary somatosensory cortex lower left leg representation validated in our prior research evaluating BOLD response to cuff inflation at the left gastrocnemius, as performed in this study (RS1_Leg_, radius 4 mm sphere centered at MNI coordinates 8, −38, 68 mm) (14). Functional connectivity maps were estimated for each participant and passed up to a second-level design contrasting CVS (*n* = 10) and healthy adults (*n* = 13) (*Z* > 2.3, *P*_corrected_ < 0.05). One healthy adult participant and three CVS participants were removed from the original dataset due to poor ECG quality. One additional CVS participant was removed from the dataset due to poor scanner localization resulting in loss of dorsal cortical data and poor normalization to MNI space near the RS1_Leg_ seed. See Table 3 for participant characteristics.

To implement individual pain sensitization as a potential explanatory variable, temporal summation indices were demeaned across groups, then split across two regressors (one for each group) as a continuous covariate to highlight functional areas where the relationships between sensitization over the course of the PAIN scan and pain-induced changes in connectivity might be different between patients and healthy adults. Significant clusters spanned multiple functional regions, a common product of cluster correction for multiple comparisons, so spherical regions of interest (ROIs; radius 4 mm) centered at local Z-statistic maxima and masked by significant clusters were generated. ROI functional region names were identified using the Harvard-Oxford cortical structural atlas, the FSL Cerebular Atlas with Nonlinear Registration, and investigator identification (25, 26). The mean connectivity signal within each ROI was extracted from each of PAIN and REST runs for each individual participant, and then differences in signal between PAIN and REST at each ROI were calculated for correlation with the temporal summation index in RStudio v2022.07.2.

### Magnetic Resonance Spectroscopy Processing and Analysis

MRSI spectra were fitted with LCModel, and metabolic maps were obtained from fitted signals. The neurochemical maps were transformed from anatomical participant space to MNI space using nonlinear registration (FNIRT, FSL) (19). A fixed-effects GLM analysis was performed evaluating group differences (CVS *n* = 6, healthy adults *n* = 10, Table 4) in Glx and GABA (FEAT, FSL, *Z* > 2.3). The average Glx signal was extracted from individual runs masked with each of the significant clusters for visualization.

## RESULTS

**Figure 2.**
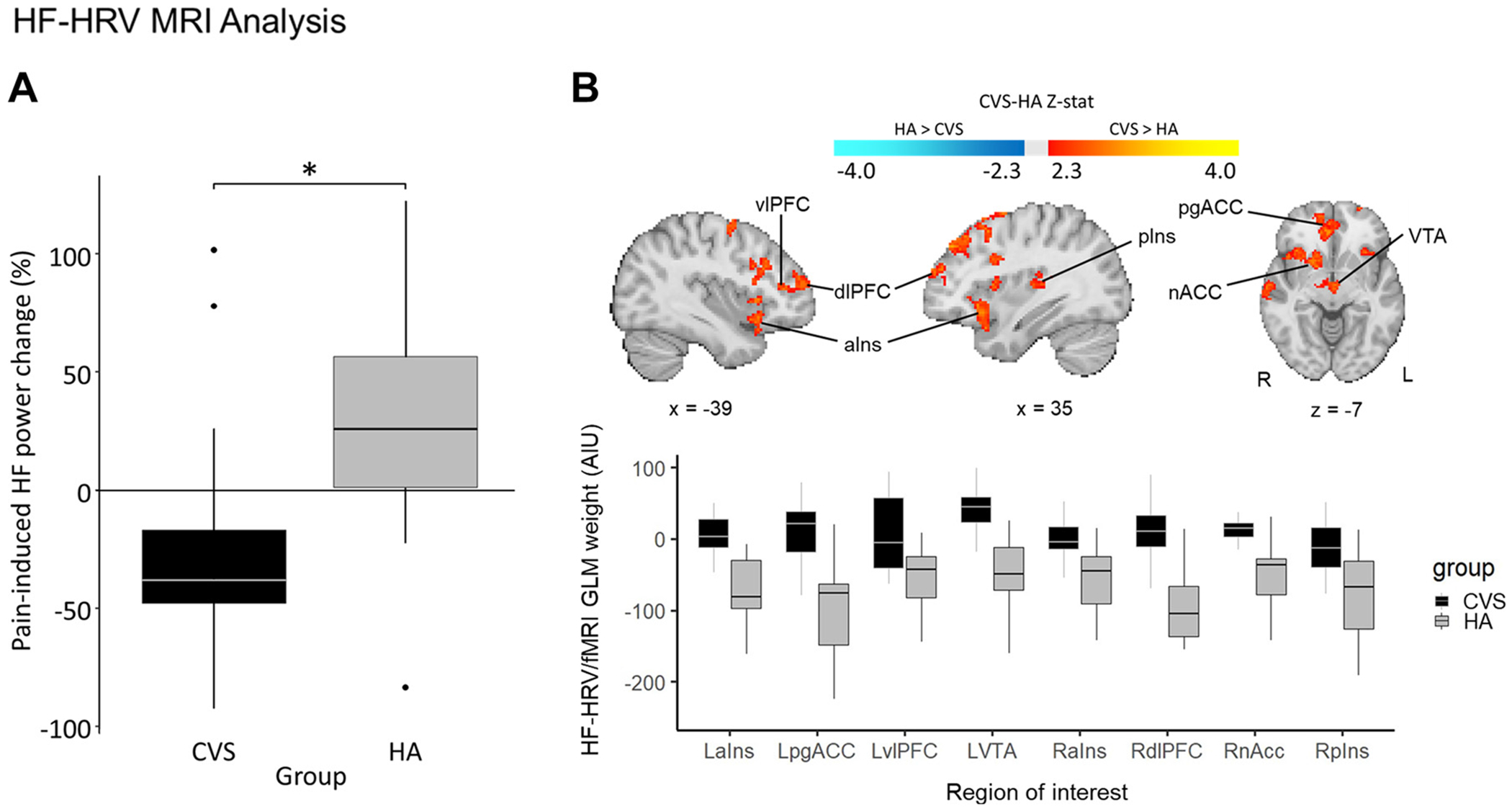
HF-HRV/fMRI response to sustained cuff pain. *A*: noxious stimulation reduced HF-HRV to a greater degree in CVS (*n* = 12) vs. healthy adults (*n* = 13; *P* = 0.02). *B*: during sustained cuff pain, patients with CVS (*n* = 13) relative to healthy adults (*n* = 13) showed reduced anticorrelation between brain activity and HF-HRV in key regions of the central autonomic network, executive control network, and dopaminergic system. No significant difference was noted at rest. *P < 0.05. aIns, anterior insula; dlPFC, dorsolateral prefrontal cortex; HF-HRV, heart rate variability within the high-frequency range; L, left hemisphere; nACC, nucleus accumbens; pgACC, pregenual anterior cingulate cortex; pIns, posterior insula; R, right hemisphere; vlPFC, ventrolateral prefrontal cortex; VTA, ventral tegmental area.

HF-HRV power normalized difference between PAIN and REST across the 6 min was significantly decreased in CVS (*n* = 12, median = −37.76, Q1 = −59.11, and Q3 = 4.66) compared with healthy adults (*n* = 13, median = 26.09, Q1 = −3.77, and Q3 = 67.03; *P* = 0.02; Fig. 2A; see Table 1 for raw HF power by group and condition). Compared with healthy adults, the CVS cohort showed weaker anticorrelation between brain activity and HF-HRV in the bilateral anterior insula, pregenual anterior cingulate cortex, ventrolateral and dorsolateral prefrontal cortex, ventral tegmental area, right posterior insula, nucleus accumbens, precuneus cortex, and thalamus during the PAIN run (Fig. 2B). No significant difference was seen between patients with CVS and healthy adult participants during REST. Table 5 shows an overview of significant functional regions resulted from the HF-HRV-driven fMRI analysis comparing CVS with healthy adults during PAIN. No significant differences in HF-HRV/fMRI were observed between groups at rest.

**Figure 3.**
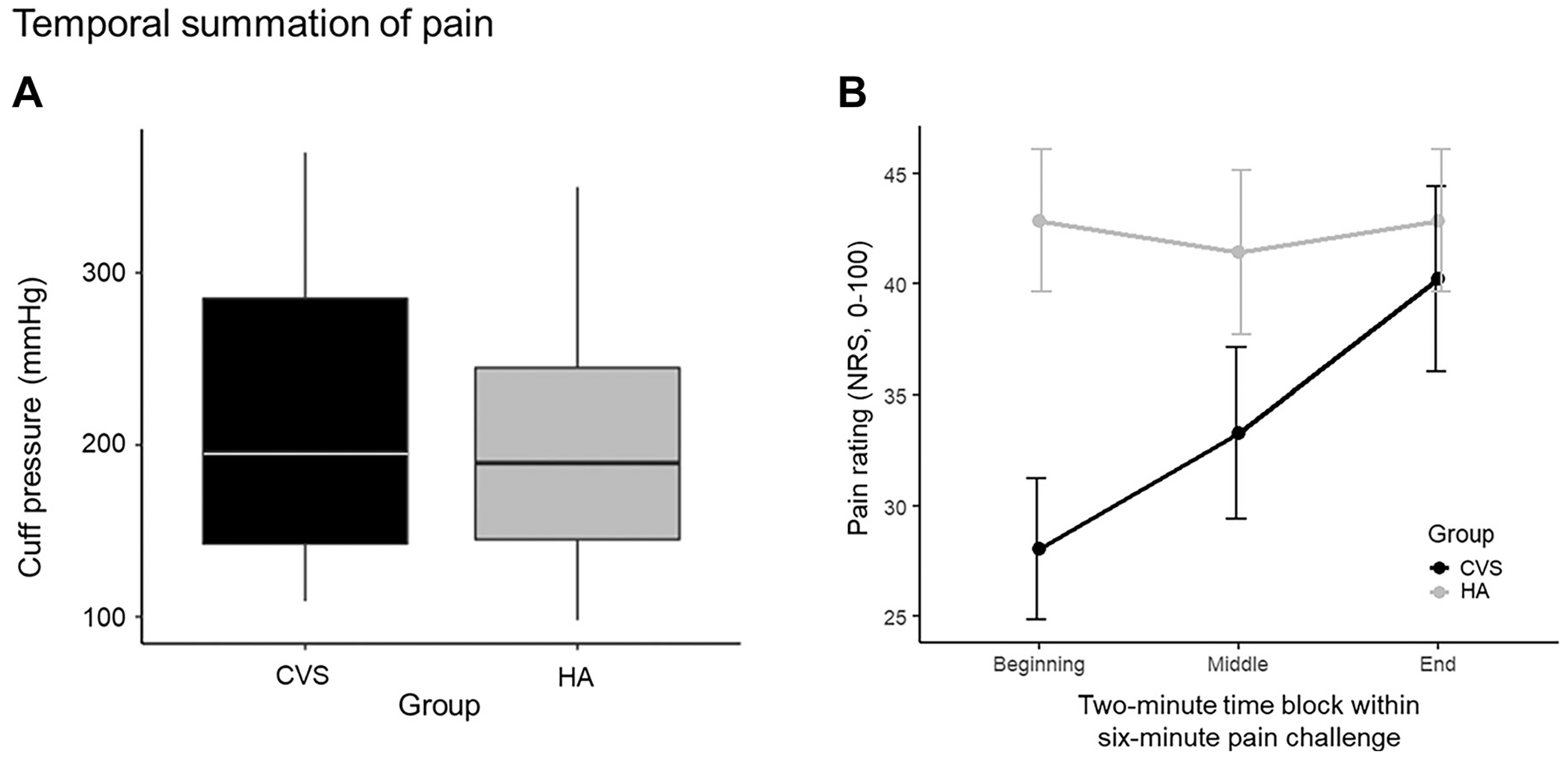
Temporal summation of pain in CVS. *A*: pressures calibrated to elicit a numeric pain rating of 40 out of 100 were not significantly different between CVS and healthy adults (HAs). *B*: paired *t* tests revealed that patients with CVS (*n* = 14), but not healthy adults (*n* = 14), exhibit significant increases in pain ratings between beginning and end 2-min blocks during 6 min of sustained pain stimulus (0–100 NRS, *P*_corrected_ = 0.003). Values are means ± SE. CVS, cyclic vomiting syndrome.

Retrospective pain ratings for the entire 6-min continuous PAIN stimulus were not significantly different between groups (paired *t* test, healthy adults: 42.14±13.11, CVS: 39.79±15.83, *P* = 0.70). A mixed ANOVA evaluating the effects of group (healthy adults and CVS) versus time (beginning, middle, and end) on pain rating revealed both a statistically significant impact of time (*F* = 7.342, *P* = 0.02), as well as a two-way interaction between group and time (*F* = 7.031, *P* = 0.02). For the CVS group, post hoc paired *t* tests with the Bonferroni correction for multiple comparisons showed a significant difference in pain ratings between the beginning (means ± SE = 28.07 ± 3.18) and end (40.21 ± 4.18, *P* = 0.003), beginning (28.07 ± 3.18) and middle (33.29 ± 3.97, *P* = 0.048), and middle (33.29 ± 3.97) and end (40.21 ± 4.18, *P* = 0.017) time periods (Fig. 3B). No significant differences were indicated between time periods within the healthy adult group. Our analysis revealed temporal summation of pain in the CVS group not shown in the healthy adults (beginning: 42.86±12.51, middle: 41.43 ± 14.34, end: 42.86 ± 12.51).

**Figure 4.**
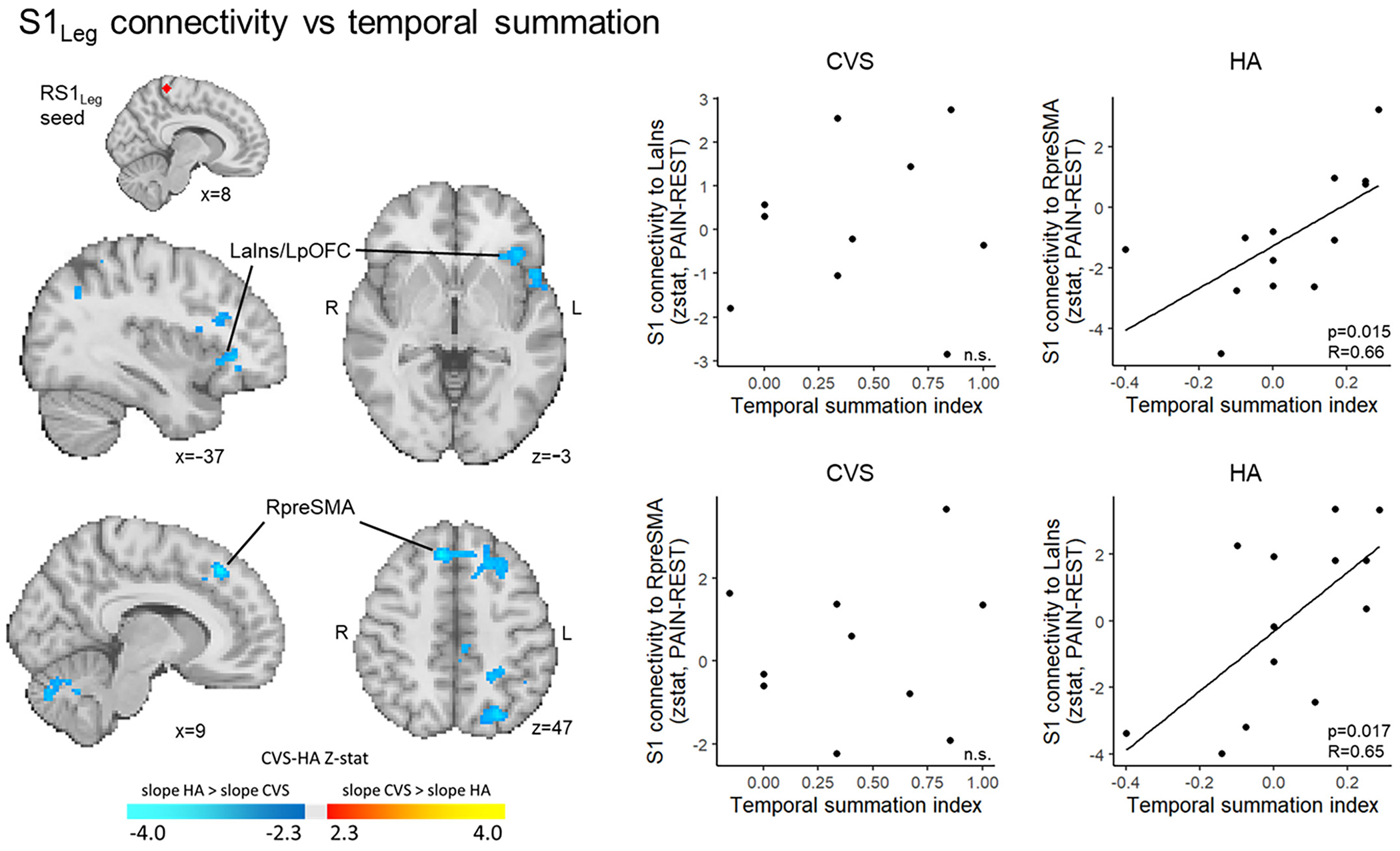
GLM analysis revealed a significant group difference in the relationship between temporal summation and pain-induced changes in S1 functional connectivity to left anterior insula (LaIns) and right presupplementary motor area (RpreSMA). Patients with CVS (*n* = 10) do not exhibit the positive correlation between temporal summation and S1 connectivity to LaIns and RpreSMA seen in healthy adults (HAs; *n* = 13). CVS, cyclic vomiting syndrome; GLM, general linear model.

GLM analysis investigating the pain-induced changes in whole brain functional connectivity of RS1_Leg_ using temporal summation as a continuous covariate highlighted areas with group differences in the relationship between temporal summation and pain-induced connectivity changes. Temporal summation was found to positively correlate with these changes in healthy adults in the left anterior insula/posterior orbitofrontal cortex (Pearson’s, *P* = 0.015, *R* = 0.66) and the right supplementary motor area (preSMA; Pearson’s, *P* = 0.017, *R* = 0.65) but not CVS (Fig. 4, Table 6).

**Figure 5.**
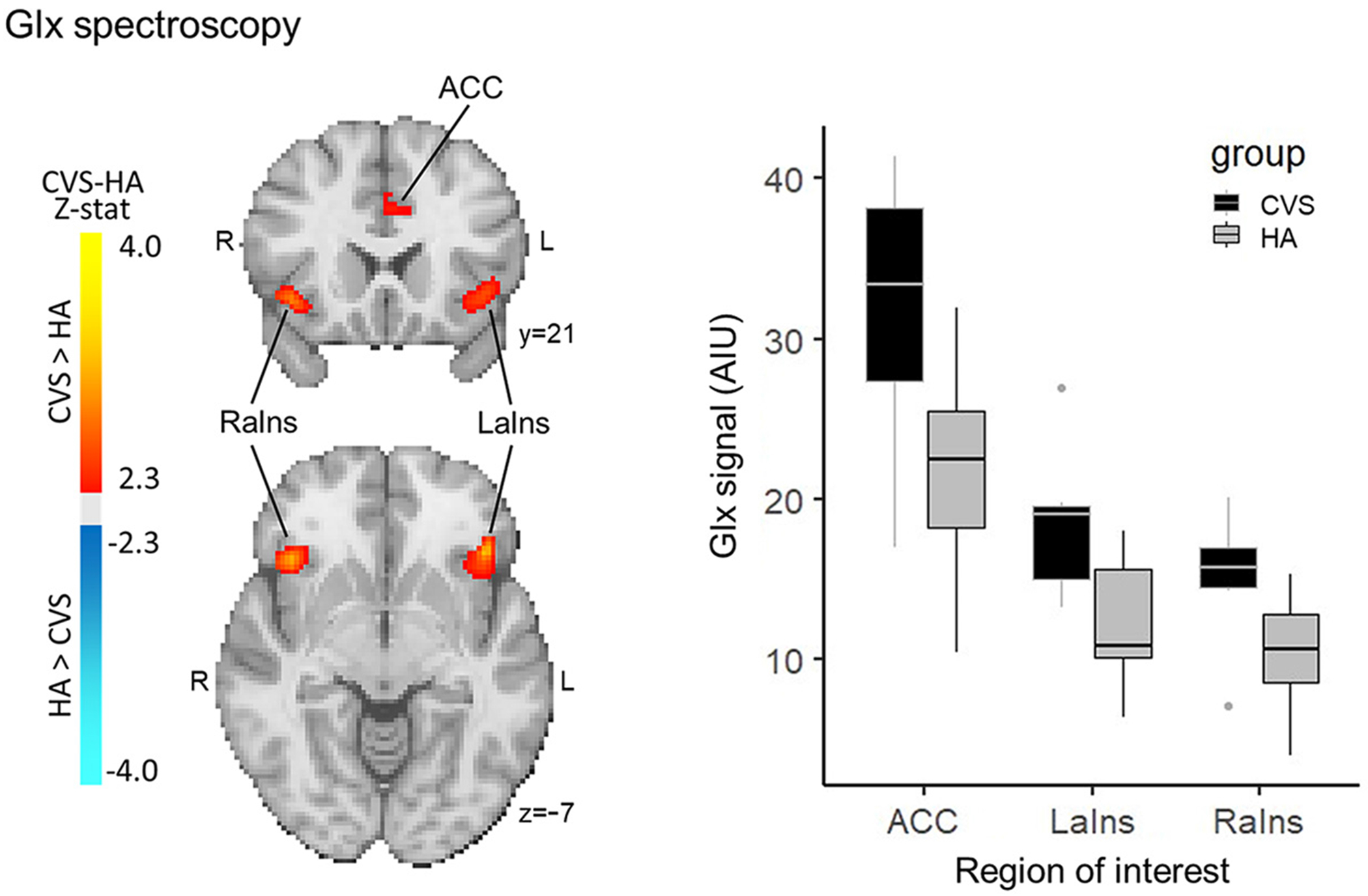
Increased Glx signal in CVS (*n* = 6) compared with healthy adults (HA; *n* = 10) without multiple comparisons thresholding in anterior cingulate cortex (ACC) and bilateral anterior insula (aIns). Subthreshold results indicate increased Glx in bilateral aIns and ACC. No differences in GABA were found between groups. CVS, cyclic vomiting syndrome.

An exploratory analysis using chemical shift imaging (MR spectroscopy; spectral editing for Glx/GABA) from a subset of our subjects (CVS *n* = 6, healthy adults *n* = 10) showed an increased Glx in regions associated with the central autonomic network, including bilateral aIns and ACC. No difference in GABA concentration was found. Although these results do not survive correction for multiple comparisons, the unthresholded map highlights the regions of increased Glx in CVS as compared with healthy adults (Fig. 5, Table 7).

## DISCUSSION

Utilizing a novel multimodal approach combining autonomic assessment with whole brain fMRI connectivity analysis, we investigated the linkage between brain response and autonomic nervous system outflow during a noxious stimulus in CVS and healthy adults. We found evidence of greater parasympathetic withdrawal (i.e., significant decrease in HF-HRV) in patients with CVS relative to healthy adults, associated with altered fMRI activity in central autonomic network regions, such as the insula cortex. Furthermore, our results showed that central sensitization was associated with aberrant connectivity from the somatosensory cortex to the anterior insula and presupplementary motor area (preSMA).

The altered parasympathetic response we report here may reflect some central mechanisms at play in CVS attacks following stressful stimuli. The significant decrease in HF-HRV found in CVS participants in response to continuous pain was not evidenced in the healthy adults and may indicate autonomic dysregulation and hyper-responsivity in the form of increased parasympathetic withdrawal, likely accompanied by sympathetic overdrive. This finding adds to prior research showing a similar correlation between the parasympathetic withdrawal and the increased brain activation among chronic pain conditions like fibromyalgia and irritable bowel syndrome (14, 27). A purported inability to maintain autonomic equilibrium in patients with CVS is also in accordance with our previous work measuring the autonomic responses to nauseogenic visual motion in patients with a propensity for motion sickness, in which we demonstrated an increase in sympathetic outflow followed by a decrease in parasympathetic outflow during nausea (10, 28). This blunted parasympathetic response is consistent with a model where stress-triggered autonomic-gastrointestinal cascade and sympathetic overdrive contribute to the nausea and vomiting episodes of patients with CVS.

Our fMRI analysis correlated this altered autonomic responsivity in CVS with functional neuroimaging outcomes, revealing a link between decreased HF-HRV and weaker autonomic/central correlation in the bilateral anterior insula and anterior cingulate cortex—regions associated with autonomic regulation and interoceptive processing (29, 30), the ventral tegmental area, and nucleus accumbens, which have been strongly implicated in the brain’s stress response (31), as well as the bilateral dorsolateral prefrontal cortex and right posterior insula, key areas involved in pain processing (30, 32). Furthermore, our nausea studies identified potential cortical inhibitors of autonomic outflow that overlap with the regions we report here, including the insula and dorsolateral and ventrolateral prefrontal cortices, suggesting a potential dysfunction of sympathetic autonomic inhibition in CVS pathology (10, 28). Taken together, these results add to the research correlating interoceptive sensations like nausea and abdominal discomfort or pain with central processing linked to a shift from parasympathetic to sympathetic activity.

Notably, we found no significant group difference between pain ratings for the entire 6-min run, suggesting that patients do not exhibit hyperalgesia in the interictal period. This result largely aligns with migraine research. Although patients with migraine are known to exhibit changes in sensory processing over the course of the episodic cycle (33, 34), recent research indicates that subsets of the patient population with higher episode frequency or chronic symptoms do not reflect increased pain sensitivity during the interictal period experienced at different stages in the cycle (35). Although variability in the extent of sensitization to the pain stimulus existed between individuals, with some participants in both patient and healthy groups reporting increasing pain over time and others decreasing pain over time, on average CVS patients, but not healthy adults, demonstrated temporal summation of pain in response to the sustained pain stimulus. We explored potential central mechanisms of this pain sensitization and the altered temporal summation of pain exhibited in patients by evaluating pain-induced changes in functional connectivity, a measure of synchronicity in activity over time, to a primary somatosensory processing brain region, S1_Leg_. In this analysis, significant correlations between pain sensitization represented by the temporal summation index and pain-induced changes in S1 connectivity with a given area implicate that area in the cognitive processing producing pain sensitization. Regions highlighted by our GLM reflected functional areas where this cognitive processing might be different between patients and healthy adults. We found that within groups, healthy adults but not patients with CVS exhibited a correlation between individual temporal summation and pain-induced connectivity changes to the left anterior insula and the right preSMA. These results suggest differential coding of pain sensitization in the salience network in CVS, where normal processing of pain sensitization or habituation is disrupted.

Altered activity and connectivity changes in the anterior insula associated with autonomic function metrics are consistent with studies implicating this region in pain-processing pathways (36). More broadly, there is a growing body of research elucidating the roles of anterior insula, posterior orbitofrontal cortex, and presupplementary motor area as key players in interoception within the wider salience network (37). Lesion studies indicate that the anterior insula is necessary for interoceptive accuracy and specificity (38), and the anterior insula and preSMA may be key regions to the prediction and processing of aversive interoceptive states (39). Primate models have recently shown that the posterior orbitofrontal cortex may play a key role in interoception processing and autonomic control (40). Future research should continue to explore this potential interoceptive dysfunction in CVS pathophysiology.

Both our autonomic- and pain-associated imaging findings highlight the potential role of the anterior insula in CVS pathophysiology. Our prior structural imaging nausea studies using diffusion tensor imaging analysis of motion sickness-susceptible and healthy adults demonstrated potential white matter microstructure differences within tracts connecting the anterior insula and the inferior fronto-occipital fasciculus, a visual motion processing region that may either contribute to nausea perception or may have resulted from a prior history of frequent nausea response (41). Furthermore, these structural changes are accompanied by increased functional connectivity between these brain regions during motion sickness (42). Evaluation of structural differences via diffusion tensor imaging in CVS might yield further insights into the functional alterations we report here.

Our exploratory analysis using magnetic resonance spectroscopy in patients with CVS found some evidence for an increased Glx signal relative to healthy adults within bilateral anterior insula and anterior cingulate, key regions of the central autonomic network. The analysis did not reveal increases or decreases in GABA, a key inhibitory neurotransmitter. GABA and Glx are thought to be balanced in the human brain (43). If the Glx signal increase is reflective of elevated levels of glutamate, acting in its capacity as the primary excitatory neurotransmitter, then these autonomic control regions might experience abnormal glutamate-mediated hyperexcitability. Therefore, future studies should further investigate a potential neurochemical mechanism underlying altered central regulation in CVS.

Our study has a few notable limitations. First, low participant counts reduced the statistical power of our analyses, particularly for H-MRS analyses. CVS patient recruitment was primarily driven by referral from a tertiary care center. Due to the low incidence of CVS and our exclusion of patients using neuromodulatory medication, limited enrollment produced a small sample size. However, this exclusion results in a much cleaner phenotype of patients being studied, not clouded by the central neuromodulatory efforts of medications. Our fMRI analyses were sensitive to noise in concurrently acquired physiological data, as well as motion artifacts, and exclusion of individual runs after failed quality checks further reduced the final sample and the statistical power of our imaging outcomes. Our magnetic resonance spectroscopy sequence was finalized after enrollment had started, and neurochemical measures were collected from only a subset of participants. Second, we did not exclude patients for lifetime cannabis or tetrahydrocannabinol use. We sought to limit the confound of potentially different pathophysiology underlying CVS versus cannabinoid hyperemesis syndrome by excluding patients who had used cannabinoids within 7 days of scanning, but more stringent standards regarding the inclusion of participants with a history of cannabinoid usage may have more effectively screened out cannabinoid hyperemesis syndrome. Finally, it is well documented that migraine participants exhibit shifts in pain perception and associated neuroimaging patterns (33, 34). Given the high degree of overlap between CVS and migraine in terms of presentation and treatment strategy, other central mechanisms might come into play during the ictal period, which could not be captured during this study due to safety concerns.

In conclusion, we integrated previous research of autonomic dysfunction in CVS with functional neuroimaging results elucidating the top-down mechanisms of interoceptive and autonomic control. Our findings indicate that the pathophysiology of CVS is characterized by multiple levels of altered physiological activity. Our results suggest that the altered autonomic response in patients with CVS might be centrally mediated. In our study, this manifested as parasympathetic withdrawal in response to a nociceptive challenge associated with a lack of an inhibitory response within key regions of the central autonomic network. Our temporal summation analysis revealed abnormal sensitization over time to the sustained pain stimulus and changes in connectivity patterns associated with that central sensitization. Finally, our exploratory analysis of potential neurochemical mechanisms in CVS pathophysiology indicated potentially increased excitatory neurotransmitter levels within regions of the brain associated with visceral sensation and interoceptive processing. Although current clinical guidelines support the use of prophylactic medication for moderate and severe CVS, the role of these medications is not yet well researched in controlled trials (8). The presence of this multilevel dysregulation in the interictal period supports the further exploration of the role and mechanism of prophylactics such as tricyclic antidepressants, which may work on the pain-processing pathways implicated here, in CVS treatment.

## Figures and Tables

**Table 1.** Characteristics of participants included in HF-HRV of analysis

	Healthy Adults (*n* = 13)	Patients With CVS (*n* = 13)	CVS vs. Healthy Adults *t* Test
Age, yr (means ± SD)	29.5 ± 11.5	26.5 ± 5.7	n.s.
Sex (number of females)	11	11	n.s.
Ethnicity	10W, 3A	12W, 1H	n/a
HF-HRV power during rest, mm^2^ (means ± SE)	1,411 ± 343	2,090 ± 687	n.s.
HF-HRV power during pain, mm^2^ (means ± SE)	1,987 ± 666	1,376 ± 474	n.s.

A, Asian; CVS, cyclic vomiting syndrome; H, Hispanic; HF-HRV, heart rate variability within the high-frequency range; W, White.

**Table 2.** Characteristics and behavioral data on subjects included in temporal summation of pain analysis

	Healthy Adults (*n* = 14)	Patients With CVS (*n* = 14)	CVS vs. Healthy Adults *t* Test
Age, yr	29.29 ± 11.08	29.21 ± 11.67	n.s.
Sex (number of females)	12	12	n.s.
Ethnicity	11W, 3A	13W, 1H	n/a
Cuff pressure for percept-matched (P40) PAIN run, mmHg	202.29 ± 81.03	222.86 ± 92.40	n.s.
Intensity of cuff pain (0–100, NRS) overall (6 min)	42.14 ± 13.11	39.79 ± 15.83	n.s.
Beginning 2 min	42.86 ± 12.51	28.07 ± 12.36	**
Middle 2 min	41.43 ± 14.34	33.29 ± 15.03	n.s.
End 2 min	42.86 ± 12.51	40.21 ± 16.23	n.s.
Temporal summation of pain intensity [(End-Beg)/Beg]	0 ± 8.55	12.1 ± 10.9	**
Unpleasantness of cuff pain (0–100, NRS) overall (6 min)	38.57 ± 20.52	38.07 ± 19.35	n.s.
Beginning 2 min	35.36 ± 15.62	25.71 ± 16.16	n.s.
Middle 2 min	36.07 ± 20.11	29.86 ± 17.55	n.s.
End 2 min	38.93 ± 19.63	39.07 ± 19.92	n.s.

Values reported as means ± SD. A, Asian; CVS, cyclic vomiting syndrome; H, Hispanic; W, White.

** *P* < 0.01.

**Table 3.** Characteristics of subjects included in functional connectivity analysis

	Healthy Adults (*n* = 11)	Patients With CVS (*n* = 13)	CVS vs. Healthy Adults *t* Test
Age, yr (means ± SD)	29.29 ± 11.08	29.21 ± 11.67	n.s.
Sex (number of females)	12	12	n.s.
Ethnicity	11W, 3A	13W, 1H	n/a

A, Asian; H, Hispanic; W, White.

**Table 4.** Characteristics of subjects included in functional connectivity analysis

	Healthy Adults (*n* = 10)	Patients With CVS (*n* = 6)	CVS vs. Healthy Adults *t* Test
Age, yr (means ± SD)	30.9 ± 12.8	26.2 ±7.5	n.s.
Sex (number of females)	9	5	n.s.
Ethnicity	7W, 3A	6W	n/a

A, Asian; H, Hispanic; W, White.

**Table 5.** Localization of significant clusters (P < 0.05) found in joint HF-HRV/fMRI analysis

Region	Label	Hemisphere	MNI Coordinates, mm			Max *z*-Score
			*x*	*y*	*z*	
Precuneus cortex	PrC	L	−1	−57	53	3.80
Dorsolateral prefrontal cortex	dlPFC	R	23	47	39	3.54
Pregenual anterior cingulate cortex	pgACC	L	−3	43	13	3.53
Nucleus accumbens	nAcc	R	17	17	−5	3.38
Anterior insula	aIns	L	−33	17	−22	3.35
Thalamus	Tha	R	1	−13	3	3.35
Ventral tegmental area	VTA	L	−1	−15	−9	3.31
Posterior insula	pIns	R	43	−15	11	3.27
Anterior insula	aIns	R	41	15	−5	3.27
Ventrolateral prefrontal cortex	vlPFC	L	−45	39	13	3.24

fMRI, functional MRI; HF-HRV, heart rate variability within the high-frequency range; MNI, Montreal Neurological Institute.

**Table 6.** Localization of significant functional regions (P < 0.05) found in seed-based functional connectivity analysis

Region	Label	Hemisphere	MNI Coordinates, mm			Max *z*-Score
			*x*	*y*	*z*	
Presupplementary motor area	preSMA	R	9	31	47	3.80
Ventrolateral prefrontal cortex	VLPFC	L	−45	27	15	3.70
Cerebellum VI lobule	CRVI	R	27	−51	−35	3.68
Intraparietal sulcus	IPS	L	−29	−69	41	3.53
Superior parietal lobule	SPL	L	−27	−49	57	3.53
Posterior midcingulate cortex	pMCC	L	−7	−21	35	3.39
S1 paracentral lobule	PCL	L	−7	−39	61	3.33
Dorsolateral prefrontal cortex	DLPFC	L	−51	7	27	3.28
Orbitofrontal cortex	OFC	L	−41	27	−3	3.24
Dorsolateral prefrontal cortex	DLPFC	L	−29	27	49	3.19
Left anterior insula	LaIns	L	−33	25	−3	2.93
Primary motor cortex	M1	L	−13	−25	75	2.83

MNI, Montreal Neurological Institute.

**Table 7.** Localization of uncorrected clusters (P < 0.05) found in Glx spectroscopy analysis

Region	Label	Hemisphere	MNI Coordinates, mm			Max *z*-score
			*x*	*y*	*z*	
Anterior insula	aIns	R	39	25	−9	3.64
Anterior insula	aIns	L	−43	27	−7	3.55
Anterior cingulate cortex	ACC	L	−3	24	31	2.55

MNI, Montreal Neurological Institute.

## Data Availability

Data will be made available upon reasonable request.
